# UBA1 enhances phagocytosis by suppressing CD47 expression in small cell lung cancer

**DOI:** 10.3389/fonc.2026.1838900

**Published:** 2026-08-20

**Authors:** Dongyun Li, Na Li, Yiqiang Liu, Junyang Chen, Zongding Wang, Lei Chen, Hong Wu, Haitao Lan

**Affiliations:** 1 Department of Oncology & Cancer Institute, Sichuan Academy of Medical Sciences, Sichuan Provincial People’s Hospital, School of Medicine, University of Electronic Science and Technology of China, Chengdu, China; 2 Kindstar Global Precision Medicine Institute, Wuhan, China; 3 Department of Hepatobiliary Surgery, Fengjie County People’s Hospital of Chongqing, Chongqing, China; 4 Department of Hepatobiliary Surgery, Fengjie Hospital, The Second Affiliated Hospital of Chongqing Medical University, Chongqing, China

**Keywords:** CD47, immunotherapy, phagocytosis, small cell lung cancer, UBA1

## Abstract

**Introduction:**

Small cell lung cancer (SCLC) exhibits a limited overall response rate to immunotherapy, largely due to its highly immunosuppressive microenvironment. CD47, an innate immune checkpoint frequently overexpressed on SCLC cells, contributes to immune evasion by inhibiting macrophage-mediated phagocytosis. Here, we uncover a previously unrecognized immunoregulatory function of ubiquitin-like modifier activating enzyme 1 (UBA1) in SCLC.

**Methods:**

We analyzed UBA1 expression in 16 SCLC patients undergoing immunotherapy (immunohistochemistry). Functional studies used UBA1-knockdown and overexpression SCLC cell lines (H446, H82) to assess phagocytosis, macrophage polarization, and CD47 regulation. Mechanistic insights were obtained via Hippo pathway activation assays (enhancing MST1/YAP phosphorylation). *In vivo* subcutaneous xenografts in nude mice evaluated tumor growth and macrophage infiltration.

**Results:**

High UBA1 expression correlated with improved immunotherapy response and prolonged progression-free survival. UBA1 activates the Hippo signaling pathway, enhancing MST1 and YAP phosphorylation and thereby repressing CD47 transcription. UBA1 overexpression attenuates surface CD47 levels in SCLC cells, potentiates macrophage phagocytic activity, and promotes a pro-inflammatory macrophage polarization, whereas UBA1 knockdown exerts opposing effects. *In vivo* xenograft models further confirm that UBA1 deficiency diminishes tumor susceptibility to phagocytosis and accelerates tumor progression.

**Conclusion:**

UBA1 enhances phagocytosis by suppressing CD47 through the Hippo-YAP pathway, improving immunotherapy efficacy in SCLC. Collectively, these findings reveal that the UBA1-CD47 axis represents a potentially pathway in SCLC and UBA1 may serve as a candidate biomarker and a potential mechanistic node for therapeutic intervention.

## Introduction

Small cell lung cancer (SCLC) is a highly aggressive neuroendocrine malignancy that accounts for approximately 15% of all lung cancer cases (1). It is characterized by rapid tumor growth, early metastatic dissemination, and profound genomic instability (2). Owing to the lack of effective early detection strategies, the majority of patients are diagnosed at an extensive stage, with a 5-year survival rate of only approximately 6% (3). Although the recent incorporation of immune checkpoint inhibitors (ICIs) into platinum-based chemotherapy regimens has provided modest survival benefits for a subset of patients, the overall therapeutic improvement remains limited, and durable responses are observed in only a minority of cases, as a result of an immunosuppressive tumor microenvironment (4, 5). These findings highlight a critical unmet need and an incomplete understanding of immune evasion in SCLC.

Tumor immune evasion is frequently mediated by the expression of “don’t eat me” signals that suppress innate immune surveillance (6–8). Among these, the CD47-SIRPα signaling axis represents one of the most well-characterized pathways that inhibits macrophage-mediated phagocytosis. CD47, a transmembrane protein widely expressed on the surface of tumor cells, interacts with signal regulatory protein α (SIRPα) on macrophages to deliver inhibitory signals that prevent phagocytic clearance, thereby promoting tumor immune escape (9). Elevated CD47 expression has been reported in multiple malignancies and is associated with disease progression and poor prognosis. Notably, emerging evidence indicates that the Hippo/YAP signaling pathway plays an important role in regulating cancer immune responses (10, 11). Specifically, when Hippo signaling is suppressed, unphosphorylated YAP translocates into the nucleus and cooperates with transcriptional partners such as TEAD to activate the transcription of various genes involved in tumor progression and immune regulation, including CD47 (12–14). However, the upstream regulatory mechanisms responsible for aberrant Hippo-YAP activation in SCLC remain largely undefined, limiting the development of effective strategies targeting this signaling axis.

In our preliminary studies, systematic screening identified ubiquitin-like modifier activating enzyme 1 (UBA1) as a potential regulator of the tumor immune microenvironment (15). While emerging studies have suggested that UBA1 is involved in diverse biological processes, including cellular stress responses, and inflammation (16–18), its role in tumor immune regulation, remains largely unexplored. Here, we demonstrate that UBA1 suppresses CD47 expression by modulating the Hippo-YAP signaling pathway, leading to reduced CD47 levels on the tumor cell surface. This process alleviates CD47-SIRPα mediated inhibition of macrophage phagocytosis and enhances antitumor immune responses. Together, these findings identify a previously unrecognized UBA1-CD47 regulatory axis that may contribute to tumor immune evasion. UBA1 may serve as a biomarker and a candidate therapeutic target, and further verification is needed.

## Materials and methods

### Reagents

RPMI-1640 medium (Gibco, A1049101); Dulbecco’s modified Eagle’s medium (DMEM, Gibco, C11995500BT); fetal bovine serum (NEWZERUM, FBS-AU500); penicillin-streptomycin (Solarbio, P1400); ampicillin (Biosharp, BL610A); glycerol (Macklin, G810584); jetPRIME Transfection Reagent (Polyplus, 101000046); Polybrene (Beyotime, C0351); puromycin (TargetMol, T19978); TRIzol reagent (Vazyme, R401-01); Evo M-MLV Reverse Transcription Kit (Accurate, AG11728); SYBR qPCR Master Mix (Vazyme, Q311-02); EndoFree Plasmid Mini Kit (Tiangen Biotech); RIPA lysis buffer (Beyotime, P0013B); BCA Protein Assay Kit (Vazyme, E112-01); SDS-PAGE reagents (Epizyme, PG112); PVDF membranes (Bio-Rad, 1620184); bovine serum albumin (Merck, B2064); ECL chemiluminescence reagent (Tanon, 180-501); red blood cell lysis buffer (Solarbio, R1010); lipopolysaccharide (Merck, L5293); interferon-γ (PeproTech, 315-05); macrophage colony-stimulating factor (PeproTech, 315-04); PKH26 dye (Sigma, MID126); CFSE dye (Merck, 21888); goat serum (Beyotime, C0265); DAB substrate kit (Maxim, KIT9901).

### Patient sample collection

This study was approved by the Ethics Committee of Sichuan Cancer Hospital and Institute, School of Medicine, University of Electronic Science and Technology of China (No. SCCHEC-04-2022-004). Written informed consent was obtained from each patient. The SCLC tissues of 16 patients were used for IHC, and the basic information is provided in **Supplementary Table 1**.

### Immunohistochemistry

Tissue samples were fixed in 4% paraformaldehyde for 48 h, dehydrated through graded ethanol, cleared in xylene, and embedded in paraffin. Sections (5 μm) were deparaffinized and rehydrated. Antigen retrieval was performed using sodium citrate buffer (pH 6.0). Sections were blocked with goat serum and peroxidase blocking reagent before incubation with primary antibodies UBA1 (1:200, Abclonal, A9254) overnight at 4 °C. After incubation with secondary antibodies for 30 min at room temperature, staining was visualized using DAB substrate and nuclei were counterstained with hematoxylin. Slides were dehydrated, mounted, and scanned using a slide scanner. Image analysis was performed using ImageJ software.

### Cell culture

Human small cell lung cancer (SCLC) cell lines H446, H82 and SHP-77 were obtained from the Chinese Academy of Sciences Cell Bank. The Human embryonic kidney cell line 293T and murine fibroblast cell line L-929 (CCL-1™) were obtained from the American Type Culture Collection (ATCC, Rockville, MD, USA). The human normal epithelial cell line HBE was sourced from Professor Zhi-Xiong Jim Xiao of Sichuan University. Cells were cultured in RPMI-1640 medium or DMEM medium supplemented with 10% fetal bovine serum and 1% penicillin-streptomycin in a humidified incubator at 37 °C with 5% CO_2_. The culture medium was replaced every two days. Cells were passaged when confluence reached approximately 80%. All experiments were performed using cells after at least three passages following thawing.

### Plasmids, shRNA sequences and overexpression constructs

All plasmids were purchased from GeneChem (Shanghai, China): *UBA1* shRNA-1 (5’-GCACAAATTAGAGATCACCAT-3’), *UBA1* shRNA-2 (5’-CCTGGGATGTCACGAAGTTAA-3’), *CD47* shRNA (5’-GCCTTGGTTTAATTGTGACTT-3’). Full-length human *UBA1* in the GV826-CMV-MCS-3flag-EF1-Luciferase-T2A-Puro vector for the overexpression assay.

### Plasmid construction, bacterial transformation, and plasmid extraction

Ligation products were transformed into DH5α competent cells. Competent cells were thawed on ice, mixed with 1 μg ligation product, incubated on ice for 30 min, heat-shocked at 42 °C for 45 s, and immediately cooled on ice for 2 min. Subsequently, 700 μL antibiotic-free LB medium was added and cells were recovered at 37 °C with shaking (200 rpm) for 1 h. The suspension was plated on LB agar plates containing ampicillin and incubated overnight at 37 °C.

Single colonies were inoculated into LB medium containing ampicillin and cultured at 37 °C with shaking (200 rpm), followed by expansion in 25 mL antibiotic-containing LB medium overnight. For glycerol stocks, 800 μL culture was mixed with 200 μL sterile 80% glycerol and stored at -80 °C. Plasmids were extracted from the remaining culture using an endotoxin-free plasmid miniprep kit. Briefly, bacterial pellets were resuspended in P1 buffer, lysed with P2 buffer, and neutralized with P4 buffer. After centrifugation, the supernatant was purified using a spin column, precipitated with isopropanol, washed with PW buffer, and eluted in 100 μL TB buffer. Plasmid concentration and purity were measured using a UV spectrophotometer and samples were stored at -80 °C until use.

### Cell transfection and lentiviral packaging

For transient transfection, shRNAs were diluted to a working concentration of 20 μM with RNase-free water and transfected using jetPRIME Transfection Reagent (Polyplus) according to the manufacturer’s protocol. Briefly, in a 6-well plate, 2 μL shRNA, 4 μL transfection reagent, and 200 μL jetPRIME buffer were mixed, incubated at room temperature for 10 min, and added to the cells. After 4 h, the medium was replaced with fresh complete medium, and cells were cultured for an additional 48 h before subsequent experiments.

For lentiviral production, HEK293T cells were seeded in 100-mm dishes and transfected at 60-70% confluence using jetPRIME Transfection Reagent with 6 μg transfer plasmid, 4.5 μg psPAX2, and 1.5 μg pMD2.G packaging plasmids. The medium was replaced 6 h after transfection. Viral supernatants were collected at 48 h and 72 h, centrifuged to remove debris, filtered through a 0.45 μm membrane, and stored at -80 °C.

For infection, viral supernatants were added to target cells (60-70% confluence) in the presence of Polybrene (8 μg/mL). After 24 h, the medium was replaced with fresh culture medium, followed by selection with Puromycin until all cells in the control group died. Stable cell lines were maintained for an additional 3–5 days before subsequent experiments.

### Flow cytometry for CD47 expression

Cells were collected and washed with PBS, then incubated with FVS700 (BD Horizon, 564997) at 4 °C in the dark for 15 min. Cells were washed with PBS, followed by Fc receptor blocking at 4 °C for 30 min. Subsequently, cells were stained with an APC-conjugated anti-CD47 antibody (Biolegend, 323123) and incubated at 4 °C in the dark for 30 min. After washing with PBS, samples were analyzed using a flow cytometer and data were processed with FlowJo. Details of the flow cytometry gating strategy are provided in **Supplementary Figure 1A**.

### Quantitative real-time PCR

Total RNA was extracted using TRIzol reagent. RNA concentration was determined using a Nanotrop2000 spectrophotometer. Reverse transcription was performed using the Evo M-MLV reverse transcription kit under the following conditions: 37 °C for 15 min and 85 °C for 5 s. The resulting cDNA was diluted to an appropriate concentration and subjected to quantitative PCR using SYBR qPCR Master Mix. Amplification was conducted under the following conditions: 95 °C for 30 s, followed by 40 cycles of 95 °C for 10 s and 60 °C for 30 s, with a final melt curve stage at 65 °C for 5 s and 95 °C for 5 min. Relative gene expression levels were calculated using the 2^-ΔΔCt method. The primer sequences used for qRT-PCR are listed in **Supplementary Table 2**.

### Western blot analysis

Total cellular proteins were extracted using RIPA lysis buffer followed by sonication. Protein concentrations were determined using a BCA Protein Assay Kit. Equal amounts of protein (25-60 μg per sample) were separated by SDS-PAGE (90 V for 30 min followed by 120 V for 1 h) and transferred onto PVDF membranes using wet transfer at 300 mA for 2 h. Membranes were blocked with 5% BSA for 1 h and incubated overnight at 4 °C with primary antibodies against UBA1 (1:1000, Abclonal, A9254), CD47 (1:1000, Proteintech, 20305-1-AP), YAP (1:1000, Cell Signaling Technology, 14074T), p-YAP (1:1000, Cell Signaling Technology, 13008T), MST1 (1:500, Abclonal, A0109), and p-MST1 (1:1000, abcam, ab76323). After washing, membranes were incubated with HRP-conjugated secondary antibody (1:5000, Beyotime, A0208) for 1 h at room temperature. Protein bands were detected using ECL Chemiluminescence Reagent and quantified with ImageJ.

### Isolation and culture of mouse bone marrow-derived macrophages

Bone marrow cells were isolated from the femurs and tibias of mice by flushing with sterile medium. The cell suspension was filtered through a 70 μm strainer and red blood cells were lysed. Cells were cultured in DMEM supplemented with 20% L-929 conditioned medium. The medium was replaced on days 2 and 5. After 7 days of differentiation, macrophages were stimulated with 240 ng/mL LPS and 20 ng/mL IFN-γ for 48 h to induce M1 polarization.

### Flow cytometric analysis of macrophage phagocytosis

Induced M1 macrophages were cultured in serum-free DMEM for 12 h and detached by trypsinization. Approximately 2 × 10^5^ macrophages were co-incubated with an equal number of CFSE-labeled H446 or H82 tumor cells in a total volume of 200 μL at 37 °C for 4 h. Cells were collected and washed with PBS, then incubated with FVS700 (BD Horizon, 564997) at 4 °C in the dark for 15 min. After incubation, cells were blocked with anti-Mouse CD16/CD32 antibody (BD Pharmingen, 553142) at 4 °C for 30 min and then stained with Brilliant Violet 421-conjugated F4/80 antibody (Biolegend, 123131) and PE-conjugated CD11b antibody (BD Pharmingen, 557397) for 30 min in the dark. Cells were washed three times with serum-free DMEM and analyzed using a flow cytometer (BD FACS). At least 10,000 macrophages were counted per sample. The phagocytic index was calculated as the percentage of CD11b^+^ F4/80^+^CFSE^+^ cells among total CD11b^+^ F4/80^+^ macrophages. Refer to **Supplementary Figure 1B** for the gating strategy.

### Laser confocal microscopy for phagocytosis assay

Bone marrow-derived macrophages were differentiated with DMEM supplemented with 20% L-929 conditioned medium for 7 days and were polarized into M1 macrophages using the same protocol. Macrophages were labeled with 20 μM PKH26 according to the manufacturer’s instructions and seeded into confocal dishes (1 × 10^5^ cells per dish) overnight. Target tumor cells were labeled with 2.5 μM CFSE. Macrophages were incubated in serum-free medium for 2 h before the addition of CFSE-labeled tumor cells (1 × 10^5^ cells). After co-incubation at 37 °C for 4 h, cells were washed extensively and observed under a laser confocal microscope (Carl Zeiss LSM 980). Phagocytic efficiency was calculated as the number of macrophages containing CFSE-positive tumor cells per 100 macrophages.

### Flow cytometry analysis of macrophage polarization

Co-culture BMDMs and tumor cells at a ratio of 1:1 in a 100mm cell culture dish for 48 hours. Cells were collected and washed with PBS, then incubated with FVS700 (BD Horizon, 564997) at 4 °C in the dark for 15 min. After incubation, cells were blocked with anti-Mouse CD16/CD32 antibody (BD Pharmingen, 553142) at 4 °C for 30 min and then stained with Brilliant Violet 421-conjugated F4/80 antibody (Biolegend, 123131), PE-conjugated CD11b antibody (BD Pharmingen, 557397) and PE-Cy7-conjugated CD86 antibody (BD Pharmingen, 560582) for 30 min in the dark. Cells were washed three times with PBS. Then, 200 μL of permeabilization buffer (BD Biosciences, 51-2090KZ) was added, followed by incubation at 4 °C in the dark for 20 min. After three additional PBS washes, cells were incubated with an APC-conjugated ARG-1 antibody (R&D Systems, IC5868A) at 4 °C protected from light for 30 min. Cells were washed three times with PBS and analyzed using a flow cytometer (BD FACS). At least 10,000 macrophages were counted per sample. See **Supplementary Figures 1C–E** for gating details.

### Protein-protein interaction network analysis

Protein-protein interaction (PPI) data was retrieved from public database of STRING. High-confidence interaction pairs were selected to construct a PPI network, where proteins were represented as nodes and interactions as edges. Network visualization and analysis were performed using Cytoscape.

### Animal experiments

Male 6-week-old BALB/c nude mice were purchased from Chengdu Dossy Experimental Animals Co., Ltd. and housed in a specific pathogen-free facility. All animal experiments were conducted at the Animal Experiment Center of Sichuan Provincial People’s Hospital in accordance with institutional ethical guidelines.

### Xenograft tumor model

H446 or H446-shUBA1 cells (1 × 10^7^ cells per mouse) were subcutaneously injected into the dorsal flank of nude mice. Tumor growth was monitored by measuring tumor length (D) and width (d). Tumor volume (TV) was calculated using the formula:


$$TV\left(mm^{3}\right)=\frac{d^{2}\times D}{2}$$

### *In vivo* macrophage phagocytosis assay

*In vivo* macrophage phagocytosis assays were performed based on previously reported methods. GFP-labeled H446 cells were subcutaneously implanted into nude mice. After 41 days, tumors were harvested and minced in PBS. Tumor tissues were digested in DMEM containing type I collagenase (1.5 mg/mL) and DNAaseI (20μg/mL) at 37 °C for 40 min to generate single-cell suspensions. Cells were filtered through a 70 μm strainer, washed three times with DMEM, and resuspended in cold flow cytometry buffer. Cells were incubated with red blood cell lysis for 10 min at 4 °C and washed in PBS twice. Cells were stained with Fixable Viability Stain 700 (BD Horizon, 564997) for 15 min at 4 °C and washed in PBS twice. Cells were blocked with anti-Mouse CD16/CD32 antibody (BD Pharmingen, 553142) at 4 °C for 30 min, followed by two washes with PBS. Cells were then blocked and stained with Super Bright 600 conjugated anti-mouse CD45 (Invitrogen, 63-0454-82), Brilliant Violet 421-conjugated F4/80 antibody (Biolegend, 123131) and PE-conjugated CD11b antibody (BD Pharmingen, 557397) at 4 °C for 30 min. Flow cytometry was performed to quantify GFP-positive macrophages, and the proportion of CD11b^+^F4/80^+^GFP^+^ cells among total CD11b^+^F4/80^+^ macrophages was calculated.

### Statistical analysis

All experiments were conducted with at least three independent biological replicates, and each assay was performed in triplicate. Data are presented as mean ± standard deviation (SD). Statistical analyses were performed using GraphPad Prism. Comparisons between two groups were analyzed using unpaired two-tailed Student’s *t*-tests. For comparisons involving three or more groups, one-way analysis of variance (ANOVA) was applied. Survival analysis was conducted using the Kaplan-Meier method, and differences between survival curves were evaluated with the log-rank test. *P* value< 0.05 was considered statistically significant.

## Results

### UBA1 expression positively correlates with ICIs response rate and survival benefit in SCLC patients

**Figure 1:**
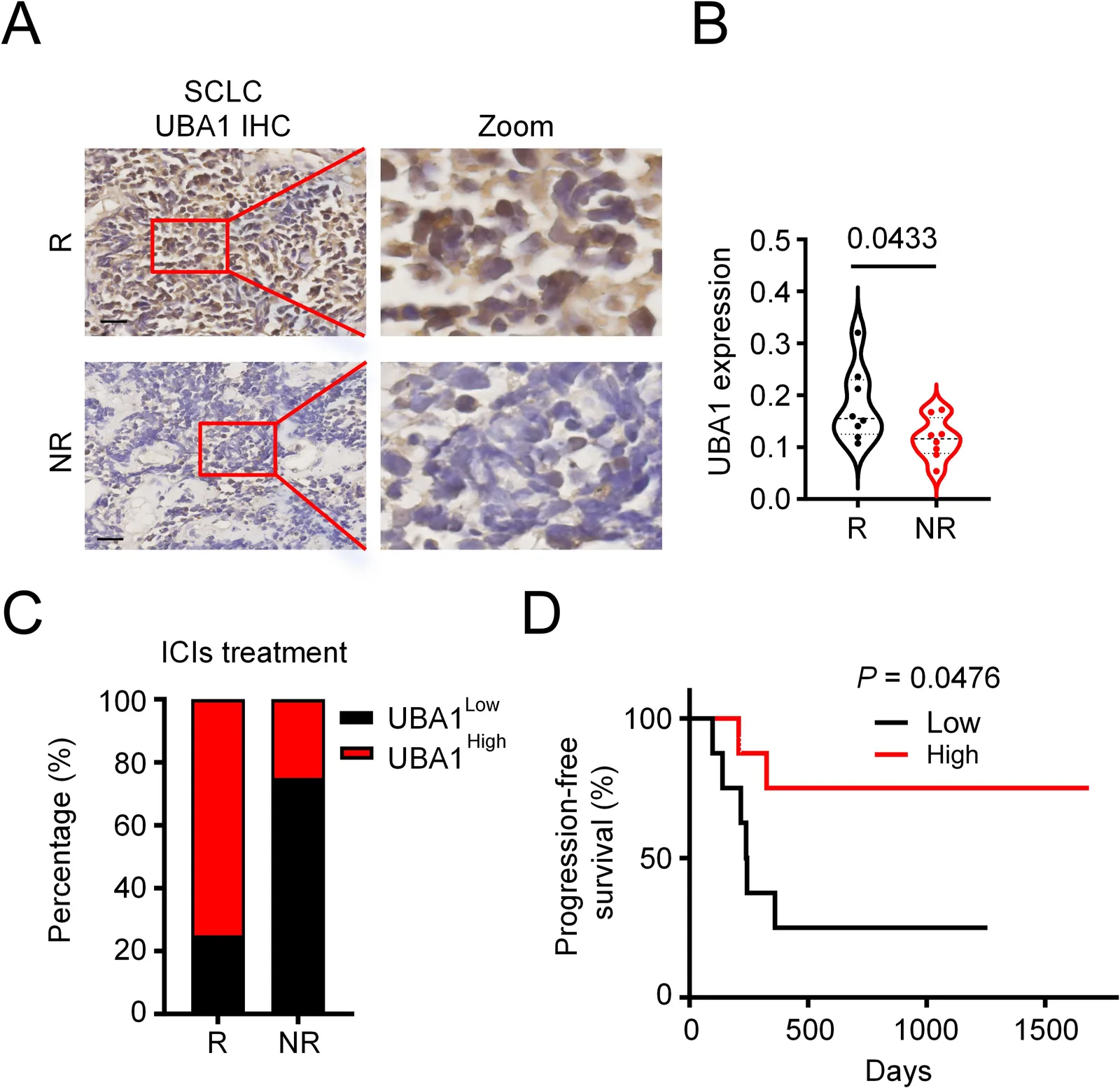
Clinical implications of UBA1 expression in SCLC. **(A)** Representative IHC images of UBA1 expression in immunotherapy responders versus non-responders with SCLC (bar: 20 um). **(B)** The expression level of UBA1 in SCLC patients (n = 16) stratified by immune checkpoint inhibitors (ICIs) response. The *P* values were calculated using Student’s t test. **(C)** The correlation between UBA1 expression and response to ICI. **(D)** Kaplan-Meier survival analysis of patients with SCLC (n = 16) based on the *UBA1* expression level. The *P* value was calculated using log-rank test.

Our prior observations established a correlation between high UBA1 expression, an inflamed tumor microenvironment, and improved prognosis in SCLC (15). Given that an activated immune microenvironment is often a prerequisite for response to immunotherapy, we hypothesized that UBA1 expression might also modulate the efficacy of immune checkpoint blockade. To test this hypothesis, we examined a cohort of 16 SCLC patients undergoing ICI therapy. We analyzed UBA1 expression levels in tumor tissues from SCLC patients treated with ICI by immunohistochemistry. Using the median of the mean optical density across all samples as the cutoff, patients were categorized into UBA1-high and UBA1-low expression groups. Our analysis revealed that UBA1 expression was significantly higher in ICI responders compared to non-responders (**Figures 1A, B**). This differential expression translated into meaningful clinical benefit: patients with high UBA1 expression exhibited a significantly higher objective response rate (ORR) and experienced prolonged progression-free survival (PFS) following ICI therapy (**Figures 1C, D**). These findings identify UBA1 as a novel predictive biomarker for both response to ICIs and favorable prognosis in SCLC.

### UBA1 promotes macrophage phagocytosis of SCLC cells

**Figure 2:**
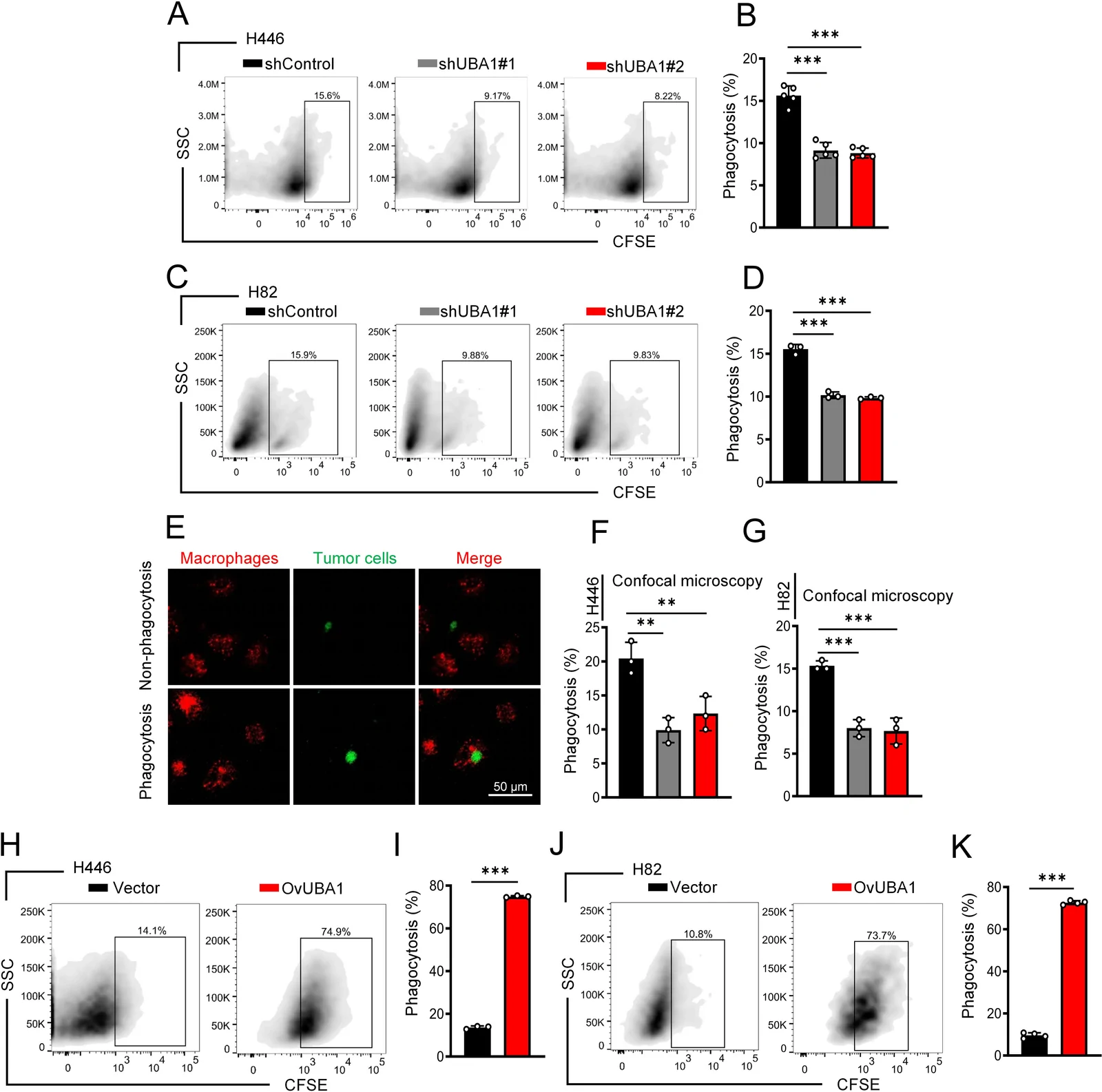
UBA1 promotes macrophage phagocytosis of SCLC cells. **(A, B)** Representative flow cytometry plots **(A)** and statistical analysis **(B)** of phagocytosis of control and *UBA1* knockdown (KD) H446 cells by bone marrow-derived macrophages (BMDMs). **(C, D)** Representative flow cytometry plots **(C)** and statistical analysis **(D)** of phagocytosis of control and *UBA1*-KD H82 cells by BMDMs. **(E)** Phagocytosis of CFSE-labeled H82 cells by PKH26-labeled BMDMs was assessed by confocal microscopy. Red, macrophages; green, targets. Scale bar: 50 μm. **(F)** Statistical analysis of phagocytosis of CFSE-labeled H446 cells with or without *UBA1*-KD by BMDMs using confocal microscopy. **(G)** Statistical analysis of phagocytosis of CFSE-labeled H82 cells with or without *UBA1*-KD by BMDMs using confocal microscopy. **(H, I)** Representative images **(H)** and statistical analysis **(I)** of phagocytosis of CFSE-labeled H446 cells with or without *UBA1* overexpression (OV) using flow cytometry. **(J, K)** Representative images **(J)** and statistical analysis **(K)** of phagocytosis of CFSE-labeled H82 cells with or without *UBA1*-OV using flow cytometry. All experiments were carried out at least in triplicate and the data are presented as the means ± SD. The P values are calculated by Student’s t test or one-way ANOVA. **P < 0.01, ***P < 0.001, ns: non- significant.

Building on our clinical observation that UBA1 correlates with an activated immune microenvironment in SCLC, we sought to explore its functional role in modulating immunotherapy response. We first confirmed the expression of UBA1 in SCLC cell lines and normal lung epithelial cells (**Supplementary Figures 2A–C**). We then established UBA1-knockdown and overexpressing derivatives in H446 and H82 cells for subsequent functional studies (**Supplementary Figures 2D–K**). These modified cells were co-cultured with bone marrow-derived macrophages (BMDMs), and phagocytosis was assessed by flow cytometry and confocal microscopy. UBA1 knockdown in both H446 and H82 cells significantly suppressed macrophage-mediated phagocytosis compared to controls, whereas UBA1 overexpression markedly enhanced phagocytic uptake (**Figures 2A–K**). Collectively, these results demonstrate that UBA1 expression in SCLC cells serves as a key determinant of their susceptibility to macrophage-mediated phagocytosis, providing a potential mechanism linking tumor-intrinsic UBA1 to an activated immune microenvironment.

### UBA1 enhances phagocytosis by downregulating CD47 via the Hippo/YAP pathway

**Figure 3:**
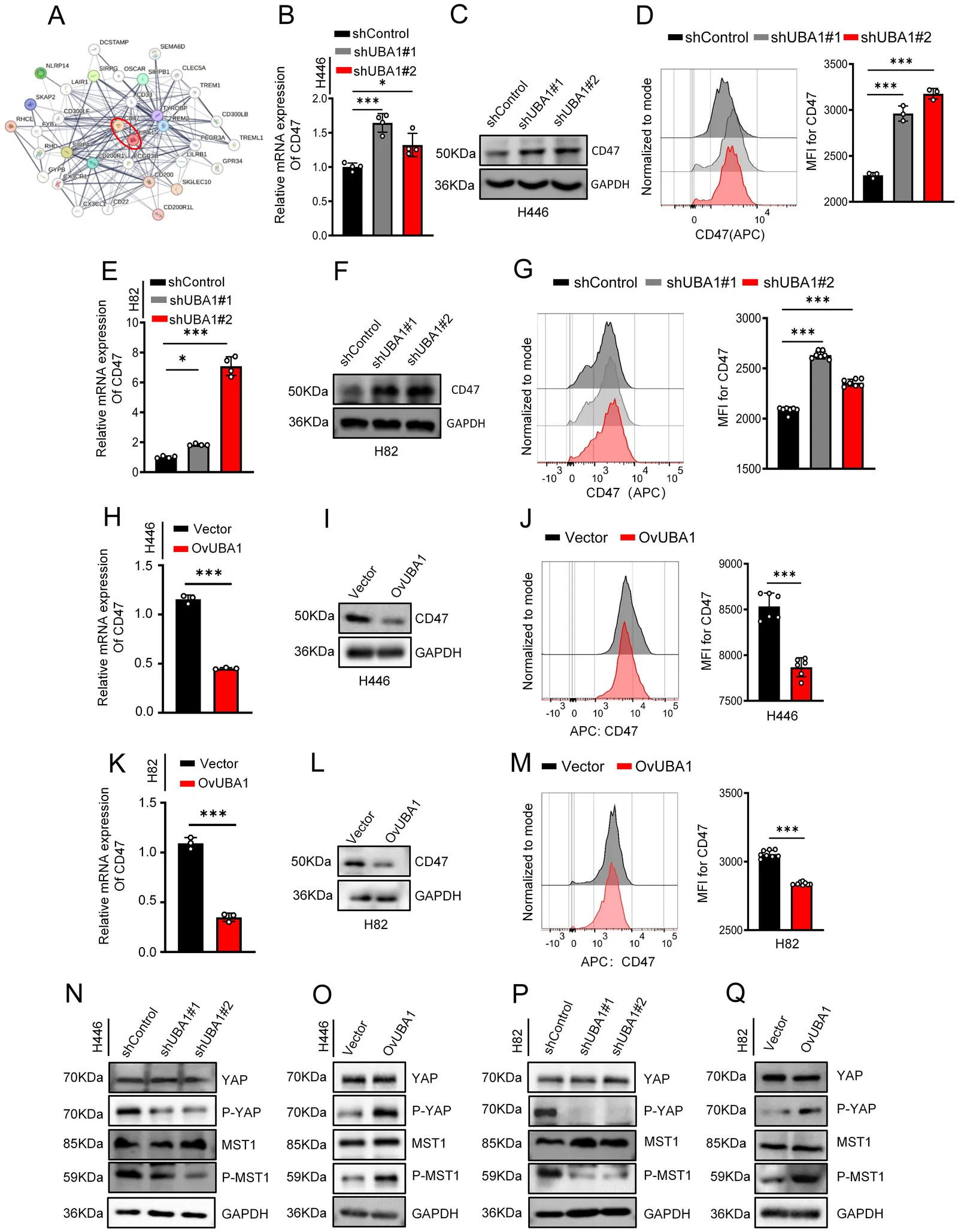
UBA1 suppresses the expression of CD47 via Hippo/YAP pathway. **(A)** Protein-protein interaction (PPI) network of UBA1. **(B–D)** The detection of CD47 expression in H446 cells with or without *UBA1*-KD by qRT-PCR **(B)**, western blot **(C)**, and flow cytometry **(D)**. **(E–G)** The detection of CD47 expression in H82 cells with or without *UBA1*-KD by qRT-PCR **(E)**, western blot **(F)**, and flow cytometry **(G)**. **(H–J)** The detection of CD47 expression in H446 cells with or without *UBA1*-OV by qRT-PCR **(H)**, western blot **(I)**, and flow cytometry **(J)**. **(K–M)** The detection of CD47 expression in H82 cells with or without *UBA1*-OV by qRT-PCR **(K)**, western blot **(L)**, and flow cytometry**(M)**. **(N–Q)** Immunoblotting analysis of indicated proteins in control, *UBA1*-KD, vector and *UBA1*-OV cells. All experiments were carried out at least in triplicate and the data are presented as the means ± SD. The P values are calculated by Student’s t test or one-way ANOVA. *P < 0.05, ***P < 0.001, ns: non- significant.

To explore the underlying molecular mechanism, we constructed a protein-protein interaction (PPI) network using the STRING database. The analysis revealed that CD47, a core “don’t eat me” signal molecule, is closely linked with UBA1 in an interaction network of 34 nodes (**Figure 3A**), suggesting that UBA1 may modulate the susceptibility of tumor cells to macrophage-mediated phagocytosis by regulating CD47 signaling. To validate this hypothesis, we examined CD47 expression in UBA1-modified SCLC cells using qRT-PCR, western blotting, and flow cytometry. The results demonstrated that UBA1 knockdown in both H446 and H82 cells led to a significant increase in CD47 expression at both mRNA levels and the protein, as well as on the cell surface (**Figures 3B–G**). Conversely, UBA1 overexpression resulted in a marked reduction in CD47 expression (**Figures 3H–M**). Given that tumor cells sustain CD47 expression through the Hippo-YAP pathway to evade macrophage recognition and phagocytosis, we next examined the protein levels within this pathway. Western blot analysis showed that UBA1 knockdown significantly decreased the phosphorylation levels of both MST1 and YAP, whereas UBA1 overexpression markedly increased their phosphorylation (**Figures 3N–Q**). Subsequently, cellular lines with dual knockout of UBA1 and CD47 were established (**Supplementary Figure 3A**). We observed that CD47 knockdown enhanced phagocytosis and also compromised the suppressive effect of UBA1 knockdown on phagocytosis (**Supplementary Figure 3B**). These results suggest that UBA1 inhibits the expression of CD47 through the Hippo/YAP pathway, thereby enhancing the phagocytic function of macrophages.

**Figure 4:**
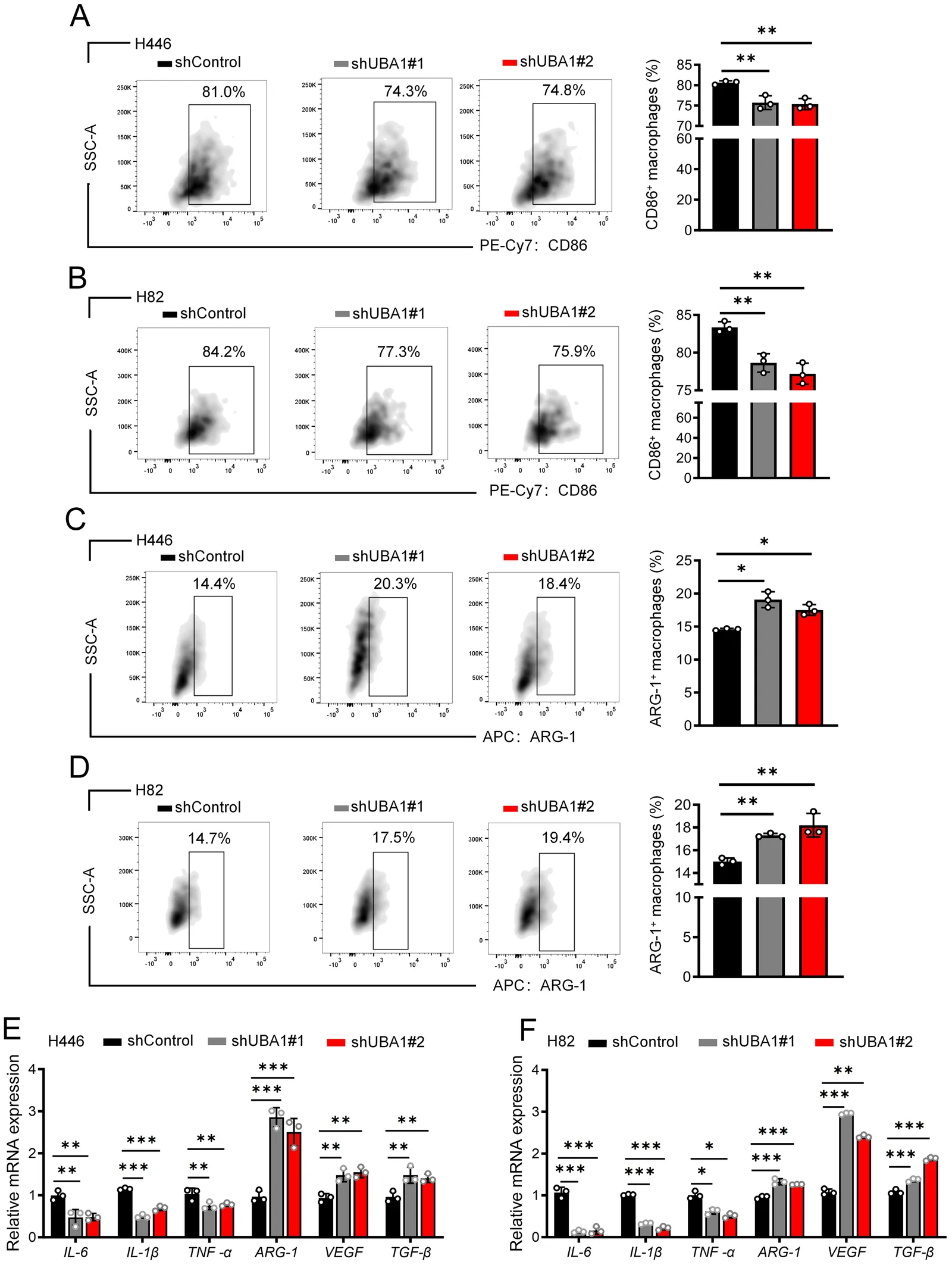
UBA1 promotes macrophage polarization toward the M1 phenotype. **(A–D)** Positive rates of M1 macrophage marker CD86 **(A, B)** and M2 macrophage marker ARG-1 **(C, D)** in control and *UBA1*-KD H446 and H82 cells by flow cytometry. **(E, F)** qRT-PCR analysis of indicated genes expression in control and *UBA1*-KD H446 **(E)** and H82 **(F)** cells cocultured with BMDMs. All experiments were carried out at least in triplicate and the data are presented as the means ± SD. The *P* values are calculated by Student’s t test or one-way ANOVA. *P < 0.05, **P < 0.01, ***P < 0.001, ns: non- significant.

To assess the functional consequences on macrophage polarization, we co-cultured BMDMs with UBA1-knockdown tumor cells or control cells. After co-culture, cells were harvested and analyzed by flow cytometry to determine the percentages of M1 macrophages (marked by CD86) and M2 macrophages (marked by ARG-1). The results showed that UBA1-knockdown tumor cells induced significantly fewer CD86^+^ M1-polarized macrophages compared to controls, while promoting a higher proportion of ARG-1^+^ M2-polarized macrophages (**Figures 4A–D**). We further assessed the expression of inflammatory cytokines in macrophages by qRT-PCR. Consistent with the phenotypic shift, BMDMs co-cultured with UBA1-knockdown cells exhibited significantly lower expression of pro-inflammatory genes (e.g., IL-6, IL-1β) and higher expression of anti-inflammatory markers (e.g., ARG-1, VEGF) (**Figures 4E, F**).

### UBA1 knockdown reduces the phagocytic susceptibility and promotes tumor growth *in vivo*

**Figure 5:**
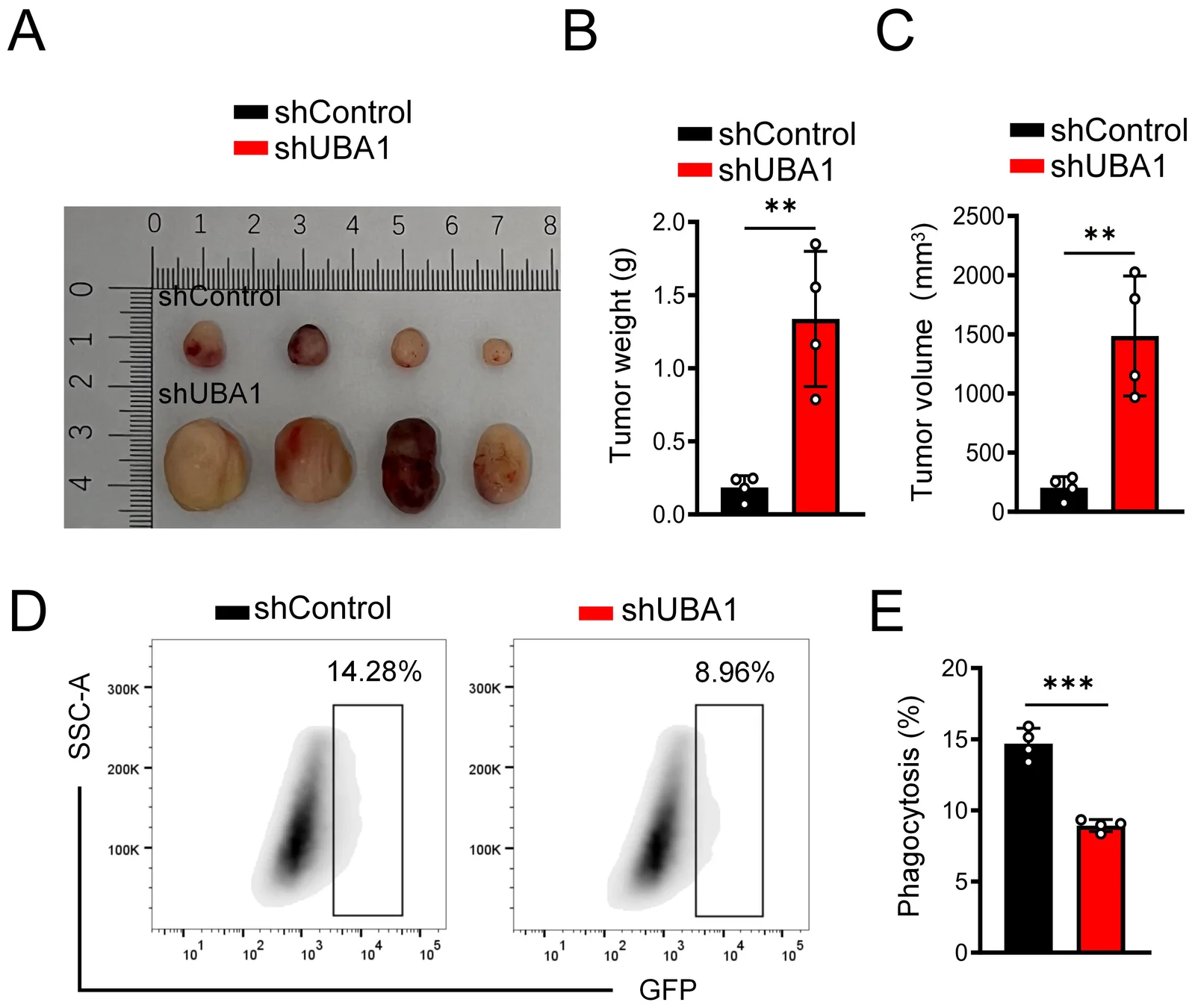
UBA1 knockdown promotes tumor growth. **(A)** An image showing the tumor tissues following subcutaneous inoculation of H446 cells with or without *UBA1-*KD. **(B, C)** Statistical analysis of tumor weight **(B)** and tumor volume **(C)** in the indicated groups. **(D, E)** Statistical analysis of phagocytosis of H446 tumor cells by macrophages within tumor tissues flow cytometric assay. All experiments were carried out at least in triplicate and the data are presented as the means ± SD. The P values are calculated by Student’s t test. **P < 0.01, ***P < 0.001, ns: non- significant.

To investigate the impact of UBA1-mediated phagocytic susceptibility on tumor growth *in vivo*, we established a subcutaneous xenograft model in nude mice using GFP-labeled H446 cells with UBA1 knockdown. The results showed that both tumor volume and weight were significantly increased in the UBA1-knockdown group compared to the control group (**Figures 5A–C**). Furthermore, flow cytometry analysis of dissociated tumor tissues was performed to evaluate the percentage of GFP^+^ macrophages among total macrophages. We found that the UBA1-knockdown group exhibited a lower proportion of GFP^+^ macrophages (**Figures 5D, E**), indicating fewer cancer cells were engulfed. In summary, these findings demonstrate that UBA1 deficiency impairs macrophage-mediated phagocytosis and promotes tumor progression *in vivo.*

## Discussion

This study highlights the pivotal role of UBA1 in sensitizing SCLC to immunotherapy. We demonstrated that high UBA1 expression in tumor tissues correlates with superior clinical outcomes in SCLC patients receiving immunotherapy. Mechanistically, we reveal that UBA1 suppresses surface CD47 expression via the Hippo-YAP pathway, thereby increasing the phagocytic susceptibility of cancer cells.

Currently, SCLC immunotherapy lacks effective predictive biomarkers to accurately identify patient subgroups that may derive significant benefits, as only a small proportion of patients achieve durable remission (19). By integrating clinical outcomes with immunohistochemistry (IHC) analysis, our study revealed that SCLC patients with high UBA1 expression exhibit higher objective response rates (ORR) and significantly prolonged progression-free survival (PFS) during immunotherapy. These findings are consistent with previous reports; however, our study uniquely reinforces this association at the protein level.

Tumor-associated macrophages (TAMs) are critical in the immune microenvironment, contributing to the metastasis, recurrence, and drug resistance of SCLC (20, 21). Although various strategies have been explored to modulate TAM infiltration and polarization, including inhibiting macrophage recruitment, repolarizing TAMs toward an anti-tumor phenotype, and identifying therapeutic targets that trigger macrophage-mediated phagocytosis (22), directly enhancing macrophage-mediated phagocytosis remains a promising yet underexplored avenue. In this study, using an *in vitro* phagocytosis assay, we demonstrated that UBA1 knockdown enables SCLC cells to evade macrophage-mediated engulfment, whereas UBA1 overexpression effectively reverses this anti-phagocytic effect. These findings suggest that UBA1 facilitates the phagocytosis of tumor cells by macrophages, though the underlying molecular mechanisms remain to be fully elucidated.

CD47 is a ubiquitously expressed transmembrane glycoprotein on cancer cells that functions as a critical inhibitor of macrophage-mediated phagocytosis (7, 23). Previous studies have established that tumor cells evade immune surveillance by upregulating CD47 to bypass macrophage recognition (24). Although therapeutic antibodies targeting CD47 have been developed, their clinical application is constrained by severe adverse effects, including anemia and thrombocytopenia (25, 26). Our study reveals that UBA1 downregulates CD47 expression, thereby triggering macrophage-mediated recognition and engulfment of SCLC cells. This process is accompanied by the release of pro-inflammatory cytokines, which inhibits tumor growth. We propose that this regulatory mechanism is associated with the phosphorylation and subsequent degradation of YAP, though the precise molecular intricacies warrant further investigation.

In summary, our study reveals that UBA1 in SCLC cells enhances macrophage-mediated recognition and phagocytosis by suppressing CD47 expression. Clinically, monitoring UBA1 levels in SCLC patients may serve as an approach for predicting the efficacy of immunotherapy and a potential mechanistic node for therapeutic intervention.

### Limitations of the study

This study has several limitations that warrant discussion. First, the clinical sample size was relatively small, which may constrain the statistical power and broader applicability of the conclusions. Future studies should expand the clinical cohort and incorporate longitudinal survival analyses coupled with multivariate Cox regression to enhance the robustness of the findings. Second, the experimental focus was primarily restricted to macrophages, and *in vivo* validation relied exclusively on a subcutaneous xenograft model in immunodeficient nude mice. Given that nude mice lack a functional adaptive immune system—particularly T cells—it remains unresolved whether UBA1-mediated phagocytosis enhancement can synergize with T cell-dependent antitumor immunity, even though the CD47-SIRPα axis is known to modulate T cell responses. Third, while we established that UBA1 regulates CD47 via the Hippo-YAP pathway, the precise mechanistic link remains incompletely defined. As the primary ubiquitin-activating enzyme (E1), whether UBA1 directly modulates the stability or activity of core Hippo components (e.g., MST1/2, YAP/TAZ) through ubiquitination requires further investigation.

## Data Availability

The raw data supporting the conclusions of this article will be made available by the authors, without undue reservation.

## Supplementary material

Supplementary Table 1 Clinical characteristics of small cell lung cancer (SCLC) patients. 1.SCLC: Small Cell Lung Cancer. 2.VALG: Veterans Administration Lung Study Group. 3.ES: Extensive-stage. 4.IHC: Immunohistochemistry.

Supplementary Table 2 The primer sequences of qRT-PCR.
