# Supplementary material for: UBA1 enhances phagocytosis by suppressing CD47 expression in small cell lung cancer
Source: Front Oncol. 2026 Aug 20;16:1838900. doi: 10.3389/fonc.2026.1838900 (PMC13539600; doi:10.3389/fonc.2026.1838900)
Supplement: Supplementary file 1 [file SupplementaryFile1.docx]

**Supplementary Figure 1**

**Figure S1. Flow cytometry gating strategy**

**(A)** Gating strategy for flow cytometry analysis of CD47 expression on tumor cell surface. **(B)** Gating strategy for flow cytometry analysis of phagocytic percentage in BMDM-tumor cell coculture. **(C-E)** Gating strategy for analyzing BMDMs polarization following coculture with tumor cells. (C) Detection of BMDMs. (D) Identification of M1 macrophages. (E) Identification of M2 macrophages.

**Supplementary Figure 2**

**Figure S2. UBA1 promotes macrophage phagocytosis of SCLC cells**

**(A)** qRT-PCR analysis of UBA1 mRNA level in SCLC cell lines.

**(B and C)** Western blot analysis of UBA1 protein level in SCLC cell lines. GAPDH serves as a loading control.

**(D and F)** Identification of *UBA1* knockdown (KD) effect in H446 (D) and H82 (F) cells using qRT-PCR analysis.

**(E and G)** Identification of *UBA1*-KD effect in H446 (E) and H82 (G) cells using western blot.

**(H and J)** Identification of *UBA1* overexpression (OV) effect in H446 (H) and H82 (J) cells using qRT-PCR analysis.

**(I and K)** Identification of *UBA1*-OV effect in H446 (I) and H82 (K) cells using western blot.

All experiments were carried out at least in triplicate and the data are presented as the

mean ± SD. The *P* values are calculated by Student’s t test or one-way ANOVA. ***P* < 0.01, ****P* < 0.001, ns: non- significant.

**Supplementary Figure 3**

**Figure S3.** **CD47 ablation rescues the phagocytosis suppression caused by UBA1 knockout**

**(A)** Identification of *UBA1*-KD and *CD47*-KD effect in H446 cells using western blot.

**(B)** Phagocytosis assay of control and *UBA1*-KD H446 cells with or without *CD47*-KD using flow cytometry.

All experiments were carried out at least in triplicate and the data are presented as the

mean ± SD. The *P* values are calculated by Student’s t test or one-way ANOVA. ***P* < 0.01, ****P* < 0.001.
