# Supplementary material for: UBA1 enhances phagocytosis by suppressing CD47 expression in small cell lung cancer
Source: Front Oncol. 2026 Aug 20;16:1838900. doi: 10.3389/fonc.2026.1838900 (PMC13539600; doi:10.3389/fonc.2026.1838900)
Supplement: Supplementary file 2 [file Table1.docx]

**Supplementary Table 1. Clinical characteristics of small cell lung cancer (SCLC) patients.**

| **Patients’**  **No.** | **Age** | **Smoke** | **Gender** | **Pathologic**  **type** | **VALG^2^** | **Using** |
| --- | --- | --- | --- | --- | --- | --- |
| 1 | 64 | Yes | Female | SCLC^1^ | ES^3^ | IHC^4^ |
| 2 | 52 | Yes | Male | SCLC | ES | IHC |
| 3 | 57 | Yes | Male | SCLC | ES | IHC |
| 4 | 51 | Yes | Male | SCLC | ES | IHC |
| 5 | 49 | No | Female | SCLC | ES | IHC |
| 6 | 78 | Yes | Male | SCLC | ES | IHC |
| 7 | 58 | Yes | Male | SCLC | ES | IHC |
| 8 | 52 | Yes | Male | SCLC | ES | IHC |
| 9 | 47 | No | Male | SCLC | ES | IHC |
| 10 | 52 | Yes | Male | SCLC | ES | IHC |
| 11 | 54 | Yes | Male | SCLC | ES | IHC |
| 12 | 46 | No | Female | SCLC | ES | IHC |
| 13 | 51 | Yes | Male | SCLC | ES | IHC |
| 14 | 64 | Yes | Male | SCLC | ES | IHC |
| 15 | 60 | Yes | Male | SCLC | ES | IHC |
| 16 | 54 | Yes | Male | SCLC | ES | IHC |

1. **SCLC:** Small Cell Lung Cancer
2. **VALG:** Veterans Administration Lung Study Group.
3. **ES:** Extensive-stage
4. **IHC:** Immunohistochemistry.
