# Supplementary material for: UBA1 enhances phagocytosis by suppressing CD47 expression in small cell lung cancer
Source: Front Oncol. 2026 Aug 20;16:1838900. doi: 10.3389/fonc.2026.1838900 (PMC13539600; doi:10.3389/fonc.2026.1838900)
Supplement: Supplementary file 3 [file Table2.docx]

Supplementary Table 2. The primer sequences of qRT-PCR.

| Name | Forward primer (3’-5’) | Reverse primer (3’-5’) |
| --- | --- | --- |
| M-IL6 | GACAAAGCCAGAGTCCTTCAGA | TGTGACTCCAGCTTATCTCTTGG |
| M-IL-1β  M-TNF-α | TGCCACCTTTTGACAGTGATG  CCCTCACACTCACAAACCAC | AAGGTCCACGGGAAAGACAC  ACAAGGTACAACCCATCGGC |
| M-VEGF | CTCGGTTCCAGAAGGGAGA | CAGCCTGGGACCACTTG |
| M-ARG-1 | GTAGACCCTGGGGAACACTAT | ATCACCTTGCCAATCCCCAG |
| M-TGF-β  M-GAPDH | CTCCCGTGGCTTCTAGTGC  GGGTCCCAGCTTAGGTTCAT | GCCTTAGTTTGGACAGGATCTG  CCAATACGGCCAAATCCGTTC |
| H-CD47 | AGAAGGTGAAACGATCATCGAGC | CTCATCCATACCACCGGATCT |
| H-UBA1 | GGACAACCACAACCACAACG | ACTTCGGACAACACGGACTG |
| H-GAPDH | GGAGCGAGATCCCTCCAAAAT | GGCTGTTGTCATACTTCTCATGG |
